# Disparate compensation policies for research related injury in an era of multinational trials: a case study of Brazil, Russia, India, China and South Africa

**DOI:** 10.1186/s12910-018-0244-y

**Published:** 2018-02-17

**Authors:** George Rugare Chingarande, Keymanthri Moodley

**Affiliations:** Centre for Medical Ethics and Law, Department of Medicine, Faculty of Medicine and Health Sciences, Stellenbosch University, PO Box 241, Cape Town, 8000 South Africa

**Keywords:** Research related injury, Compensable injuries, No fault compensation, Exclusions

## Abstract

**Background:**

Compensation for research related injuries is a subject that is increasingly gaining traction in developing countries which are burgeoning destinations of multi center research. However, the existence of disparate compensation rules violates the ethical principle of fairness. The current paper presents a comparison of the policies of Brazil, Russia, India, China and South Africa (BRICS).

**Methods:**

A systematic search of good clinical practice guidelines was conducted employing search strategies modeled in line with the recommendations of ADPTE Collaboration (2007). The search focused on three main areas namely bibliographic data bases, clinical practice guidelines data bases and a restricted internet search. A manual search of references cited in relevant guideline documents was also conducted. The search terms, Medical Subject Headings (MeSH) and key words were developed for a PubMed platform and then adapted for all other data bases. The search terms were kept constant for each country with the only difference being the country name. The documents so obtained were subjected to systematic content analysis.

**Results:**

The study revealed that there is vast panoply of regulations which exist on a continuum. On one extreme is India with comprehensive regulations that are codified into law, and on the other end there is China which does not have specific laws regulating research related injuries. There are a number of differences and similarities such as mandatory insurance requirements, existence of no fault compensation, compensable injuries and the role of research ethics committees.

**Conclusions:**

It is imperative to enact legislations that protect participants without stifling the research enterprise. There is need for consistency and ideally harmonization of such regulations at a global level. A model policy on compensation for research related injuries should borrow from the best aspects of the different country policies and should be informed by the cardinal ethics principles of autonomy, justice and beneficence.

## Background

Extant literature is replete with examples of research related injuries, and fatalities. The deaths of research participants Ellen Roche and Jesse Gelsinger [1] received widespread coverage, both in the media and in academia. The near disastrous consequences of a clinical trial of the humanized monoclonal antibody (TeGenero) TGN1412, complications associated with egg donation for stem cell research and many other major adverse events have also received attention [1]. All these, separately and collectively, bring the question of research injury compensation into sharp focus.

The moral argumentation for the compensation of research injury is premised on three ethical principles namely beneficence, justice and reciprocity [2]. The principle of beneficence dictates that the benefits of research should outweigh the risks. Hence the risks should be minimized [3–5]. Risk minimization is two-fold. First, it captures the need to reduce the risk of conducting research itself. Second, when injury does occur researchers have an obligation to mitigate the health effects as well as other effects such as economic loss. The justice principle encapsulates both distributive and compensatory justice. Distributive justice requires that the benefits and risks accrued in the research enterprise should be equitably carried by the research participants and the society. The participants bear disproportionately the burdens of participating, and therefore the burdens associated with mitigation of research related injuries should be apportioned to society as a means of equalizing the scales. Undergirded by these ethical principles a moral consensus has emerged in favour of compensation for research related injuries. This consensus is captured in several documents [6–9].

The emerging ethical consensus in favour of compensation for research related injuries has not been operationalized into standard international policies and regulations. In the absence of specific policies, standards and regulations for injured participants; those desirous of seeking redress must rely on TORT law. This poses a serious problem to injured research participants because tort law, in contradistinction to no fault compensation principles, generally requires the aggrieved party to prove that the one alleged to have caused harm was at fault. After this threshold, has been met, three further requirements should be satisfied. First, that the researcher did not fulfill a duty owed to the participant; second, that the researcher’s failure to fulfill that duty caused the participant’s injury; and third, that the researcher did not have legal justification for the failure [10]. The very nature of research means that injured research participants will have difficulty proving each of these elements [11]. A good informed consent form clearly spells out the duty owed by the researcher to the participant. Research related injuries could still occur in spite of the researcher diligently fulfilling his obligations. In such a case, the tort system absolves the researcher. Even in the case where the researcher fails to fulfill the duty owed the participant, the participant must still demonstrate a causal link between the failure and the injury. Demonstrating this is not easy especially considering the fact that in some conditions it is not feasible to trace the occurrence of an injury to a single event. For example, how does one demonstrate that a particular cancer was caused by a specific failure on the part of the researcher when cancer is known to occur in the general population not participating in the research? Where failure is proved and a causal link is demonstrated, if the researcher can proffer a legal justification for the failure, the case falls on this.

Some research related injuries arise from totally unforeseen risks. Since these risks are unforeseen, they cannot be addressed in the informed consent process. The researcher cannot also not be faulted for their occurrence. Such injuries, therefore, fall outside the purview of the tort system leaving the injured participant with no recourse.

The research enterprise is expanding phenomenally as is evidenced by the fact that the number of clinical trials registered on the United States website clinicaltrials.gov increased from 5636 in 2000 to 167,456 by May 2014, of which more than 51% were being conducted at sites outside the United States [12]. The globalization of the clinical trials industry has seen countries such as Brazil, Russia, India, China and South Africa (BRICS) become regional hubs of clinical trials. Furthermore, it was estimated that, approximately 30% of the global contract research market supporting clinical research activities, including research and development of pharma industries would be outsourced to developing countries by 2008 [11]. Research related injuries in these countries are significant. For example, documents presented to the Supreme Court in India in 2013 revealed that between 2005 and 2012 as many as 2868 participants had died during trials of which only 82 had been compensated [13]. This necessitates an investigation into these countries’ research related injury policies.

The globalization of the research enterprise is happening at a time when there are no international standards on compensation of research related injuries. Presently, the United States has no national standards or policy on compensation of research related injuries [14] and yet it is one of the major sources and sponsors of global research. The European Union is guided by the European Regulation no 536/2014 on clinical trials on medicinal products for human use. Disparate standards create problems in the resolution of injury claims which, in a globalized industry, will likely emanate from different countries. It becomes imperative for sponsors of global research to familiarize themselves with local standards before they choose the destinations of their research activities. The policies and regulations of the BRICS, which is a major destination of global research, naturally become a primary concern. Imagine a hypothetical scenario involving five adverse events leading to death in a multi-center clinical trial conducted in the BRICS countries, with one trial related fatality occurring in each country. The sponsor is faced with five different conundrums and the quantum of compensation, all things being equal, if any, will be different in each case. A system in which participants who suffer similar injuries receive differential compensation patently violates the ethical principle of fairness.

This problem is further exacerbated by the fact that under United States (U.S.) federal regulations, U.S. sponsors, who constitute a significant contributor in terms of both volume and number to the global research enterprise, particularly the (National Institutes of Health) NIH, are not required to provide compensation for the treatment of research-related injury for trial participants or to allow grant funds to be used by investigators for appropriate insurance [15]. This poses a serious challenge in developing countries where most participants have no access to health insurance. The absence of adequate compensation for research related injuries places an extra strain on already poorly resourced healthcare systems in the developing world.

The primary objective of this study was to perform a comparative analysis of the compensation for research-related injury policies of the BRICS countries and propose a more protective model for the insurance and compensation of the injured research participants. The secondary objectives were as follows:

- To determine whether there are voluntary or compulsory requirements to obtain insurance for research participants in each country.
- To identify the kind of compensable injuries with emphasis on death, serious harm, pain suffering and economic losses.
- To determine whether there are any exclusions spelt out in policies
- To identify the actors responsible for providing compensation
- To determine whether no- fault compensation is provided for in cases where negligence cannot be established.
- To identify the actors responsible for adjudication and their responsibilities.
- To establish the roles of Research Ethics Committees (RECs) in compensation for research related injuries.

## Methods

Guided by the research objectives a systematic search of good clinical practice guidelines was conducted employing search strategies modeled in line with the recommendations of ADAPTE Collaboration (2007) [16]. The search focused on three main areas namely bibliographic data bases, clinical practice guidelines data bases and restricted internet search. A manual search of references cited in relevant guideline documents was also conducted. The list of data bases consulted is contained in Table 1 below. The search terms, Medical Subject Headings (MeSH) and key words were developed for a PubMed platform and then adapted for all other data bases. The search terms were kept constant for each country with the only difference being the country name. The following search terms were used for all the data bases: research related injury compensation policies; medical related injury compensation; clinical trial related injury compensation; compensable injuries; exclusions. For some data bases extra search terms were used. These are shown in Table 1. The internet search revealed that the Office of Human Research Protection (OHRP) [12] annually publishes a compilation of international human research standards that covers the policies and standards for all the countries in the current study. The documents found on this website were identical with the documents found from other sources. Documents that were not in English were translated using both translate.google.com and freetranslate.com. These translations were then compared to the English versions found on the OHRP website. The documents so obtained were subjected to systematic content analysis. For each country information on seven variables was collected. These variables are: the existence of a written policy, insurance policy requirements, compensable injuries, exclusions, and actors responsible for compensation, existence or absence of no-fault provisions, actors responsible for adjudication of claims and the roles of the RECs. Although there were grammatical differences between the translated documents; there were no differences in the content of the variables of interest. All other relevant information not captured by these variables was also gathered and categorized.

**Table 1:** List of data bases and search terms

Data Base	Location	Extra Search Terms
CINAHL	www.ebscohost.com	
Embase	www.embase.com	
Web of Science	Wokinfo.com	
PubMed	www.ncbi.nlm.nih.gov/pubmed	
National Guidelines Clearinghouse	www.guidelines.gov	Guidelines; policies; recommendations; compensable injuries
Guideline International Network Library	www.g-i-n.net	Guidelines; policies; recommendations; compensable injuries;
National Library of Medicine	www.nlm.nih.gov	Guidelines; policies; recommendations;
National Institute for Health and Clinical Excellence	www.nice.org.uk	Guidelines; policies; recommendations;
Google	Google.com	Guidelines; policies; recommendations;

## Results

The results are summarized in Table 2 below.

**Table 2:** Summary of findings

Country	Written Policy	Voluntary insurance/Compulsory	Compensable injuries	Exclusions	Actors responsible for compensation	No fault compensation	Ajudicator	Legal enforceabi-lity
Brazil	Yes	No	All injuries; death	Failure to prove negligence	Sponsor Investigator Research Institution	No	Courts	Yes
Russia	Yes	Yes	Death, 1st, 2nd, 3rd degree disability, none disabling injuries	Economic losses Psychological injuries	Sponsor	No	Insurance Companies	Yes
India	Yes	No	All injuries including non trial related and economic losses	No	Sponsor	Yes	The Licensing Authority; REC and Expert Committee	Yes
China	No	No	Product quality liability; Drug administration errors	Injuries due to medication and procedures that meet minimum national and industry standards	Sponsor	No	None	No
South Africa	Yes	Yes	Bodily injuries Enduring injuries	Psychological injuries; non- enduring injuries	Sponsor	Strict liability	Courts	No

### Brazil

In Brazil research-related injuries are covered by resolution 196/96 of the Brazilian parliament on research involving human subjects sections V5 to V7 which states that:

*“* ***V.5*** *The researcher, the sponsor and the institution must assume full responsibility for providing comprehensive care to the research subjects, as regards complications and injury resulting from foreseen risks.”*

***“V.6*** *Research subjects that suffer any type of injury resulting from their participation in the research, regardless of such injury having been foreseen in the terms of consent, or not, have the right to receive comprehensive medical care, as well as an indemnity.”*

***“V.7*** *Under no circumstance will the research subject be required to waive his/her right to indemnity for injury resulting from the research. The form used in obtaining the freely given and informed consent of the research subjects must not contain any clause exempting the researcher from responsibility or depriving any individual of his/her legal rights, including the right to seek an indemnity for injury resulting from the research.”* (National Health Commission,2014)[17]

Furthermore, injury is defined as *“****Injury associated to or resulting from research -*** *immediate or delayed injury to an individual or community, with proven, direct or indirect, causal relationship resulting from the scientific study while* ***Indemnity –is*** *financial compensation provided as a reparation of immediate or delayed injury caused by research to a human subject of such research.”*

Premised on this resolution Brazilian law does make provision for compensation but does not make it compulsory for sponsors of research to provide insurance prior to commencement of trials. This compensation comes in two forms. First, there is “comprehensive medical care” designed to ameliorate any injury or complications resulting from participation in research. This injury, per the definition, can be immediate or delayed. This suggests that medical cover should include cover for late effects. However, the regulations do not specify a time limit for the delayed injuries. Some injuries such as some radiation effects or some genetic effects have relatively high latency. Hence, the silence on the time within which claims can be made is a weakness in the regulations from the sponsor’s viewpoint. Furthermore, the resolution is unambiguous in identifying that “the sponsor, the investigator and the institution” assume full responsibility for medical care. However, it does not specify whether they are severally or individually liable and in what proportion they assume this responsibility.

The second form of compensation is “indemnity”. This is financial compensation provided as reparation of injury which can either be immediate or delayed. To qualify for indemnity the research participants must have suffered any type of injury. The implication is that both physical and psychological injuries are covered. The resolution does not make mention of economic losses; therefore, it can be surmised that the drafters of the resolution did not intend to include such losses.

The silence of the regulations on the issue of economic losses is a disadvantage to the participant. Clarity on this issue helps inform the prospective participant’s decision. In the absence of cover for economic losses the participant has an option to take the risk and enroll on the research without insurance cover or to explore alternative insurance arrangements. Placing the burden of economic losses, which can in some cases be substantial, on the shoulders of the participant is a double burden The participant must not only assume the risk of participating in an enterprise meant for the benefit of society, but also carry the burden of arranging at the participant’s cost insurance against economic loss. The society on its part does nothing but reap the benefits of the participant’s benevolence. This patently violates the principle of justice.

The resolution does not prescribe the path to be followed by aggrieved research participants. It also offers no guidance in the determination of the quantum of the indemnity. In the absence of a clear prescription on the path to be followed the aggrieved participants would have to seek recourse in the civil courts, which can prove to be burdensome. Furthermore, whereas the resolution is clear that the sponsor, the investigator and the institution are responsible for comprehensive medical cover, it is silent on who should be responsible for the indemnity. The assumption is that the same parties would shoulder the responsibility of indemnifying the injured participants. Who should the aggrieved research participant approach? Should such a participant join all the parties in the civil action in the event of dispute?

The resolution makes no reference to “no fault” compensation. However, the prosaic definition of reparation is “***the action of making amends for a wrong one has done, by providing payment or other assistance to those who have been wronged.***” Indemnity as defined in Brazilian law is “a reparation” and reparation presupposes that wrong has been done by the one paying the reparation. The indemnity provision, therefore does not meet the basic dictates of the “no fault” compensation principle. The law goes on to state that “*Under no circumstance will the research subject be required to waive his/her right to indemnity for injury resulting from the research.”* This provision caters for all the different scenarios that can result in injury including incidences involving the participant’s actions contributing to his harm.

Although the role of the REC is not explicitly enunciated, the law stipulates that the informed consent form “*must not contain any clause exempting the researcher from responsibility or depriving any individual of his/her legal rights, including the right to seek an indemnity for injury resulting from the research”* Hence RECs should study protocols carefully and ensure that they do not violate this provision.

### Russia

In Russia, research- related injuries are governed by ***the*** Russian Federation Law №61-FZ March 24, 2010 On Circulation of Medicines. Chapter 7 Clinical Trials of Medicinal Products for Medical use, Clinical Trial Contract, Rights of Patients Involved in Trials - Article 44. Compulsory Insurance of Life and Health of the Patient Involved in Clinical Trial of Medicinal Product for Medical Use***.***

The law mandates the institutions that have acquired permission to conduct clinical trials to obtain compulsory insurance for all research participants. The insurance covers the life of the participant as well as health impairment. The restriction of the insurance to the life of the participant precludes cover of babies injured in utero. Health impairment is further categorized into four groups namely first-degree disability, second-degree disability, third degree disability and harm that does not cause disability. The law further stipulates the quantum of the damages to be paid as follows: death (Russian Rupees) RUR 2.0 million; first degree disability RUR 1.5 million; second degree disability RUR 1.0 million; third degree disability RUR 500,000 and not more than RUR 300,000 for harm which does not cause disability. To put these figures into context, it is worthy highlighting that according to Rosstat the average annual income of a Russian worker was 257,000 Russian Rupees in January 2016 [18]. The compensation for harm that does not cause disability is therefore more than the average annual income.

The law offers no guidance on how disputes over the degree of disability are to be resolved. There is a provision in the law which allows the courts to increase the quantum of the compensation, but circumstances that merit an increase of the quantum of compensation are not spelt out.

The term of the insurance cover shall not be less than the term of the clinical trial and standard insurance terms and conditions apply. Since standard insurance contract rules normally do not pay when the insured person is at fault, injured research participants whose injuries arise because of non-compliance or negligence on their part may not be compensated. This scheme is thus not a no fault scheme, making it fall short of the minimum required ethical standard.

The claims should be satisfied within the time limit prescribed by the law for civil litigation. Thus, delayed effects linked to the research but appearing after the prescribed time are not claimable under this law. Once the necessary documentation has been presented the insurer is obliged by law to make payment within thirty days. There is also a provision for the insurer to make partial payment at the request of the affected participant while the full extent of the harm is being established. The partial payment covers the harm that has already been established definitively. It is the responsibility of the federal executive body which issues permits for clinical trials to make sure that the institutions conducting clinical trials have obtained the requisite insurance. The process to be followed to make claims is not described save to say that standard terms applying to compulsory insurance shall apply.

The law is silent on the issue of “no fault” compensation. However, it refers to the application of “standard rules for compulsory insurance.” Such rules are normally stringent and restrictive. The law provides for insurance to be put in place for the life and health of the participant and does not extend to cover economic losses. When economic losses are substantial and devastating the scales of distributive justice are tipped against the participant.

Psychological injuries are not mentioned directly but may possibly be covered under injuries that cause no harm. However, the quantum of compensation for such injuries is capped at RUR 300,000, which is the lowest. This suggests that injuries that cause no physical harm are less debilitating than physical injuries. Clearly, this is not the case as some physical injuries are of a transient nature while some post stress disorders can be enduring. The role of the REC, with regards to compensation, is to ensure that sponsors of research have made adequate insurance arrangements.

### India

In India, the compensation for research related injuries is governed by the Drugs and Cosmetics Rules. This law specifically deals with clinical trials. A raft of amendments was made to the rules in 2013 in response to perceived exploitation of research participants. Documents presented to the Supreme Court in India in 2013 revealed that between 2005 and 2012 as many as 2868 participants had died during trials of which only 82 had been compensated [13]. The high mortality coupled to the very small proportion of compensated participants gave impetus to the new legal regime.

The law lucidly spells out several issues. First, it refers to injury occurring to clinical trial subjects, and then injury occurring to clinical trial subjects that is related to the clinical trial. The major import of this provision is that a distinction is made between injuries related to the clinical trial and injuries not related to the clinical trial. However, both types of injuries are to be compensated. The compensation of injuries not related to the research is unique in compensation literature. For the avoidance of disputes, the law states that, “In case of injury or death of a person in course of a clinical trial, whether such injury or death has been caused due to the clinical trial, shall be decided by the Drugs Controller General of India or such authority in such manner as may be prescribed.”

Secondly, the law makes a further distinction in the approaches to the compensation of the two types of injuries. For the injury that occurs during a clinical trial but is not related to the trial compensation takes the form of comprehensive medical care for as long as is necessary. For the injuries that are related to the clinical trial, over and above comprehensive medical treatment the injured participant or his nominees also receive a financial compensation in the event of death.

Third, the power to determine the quantum of the compensation is reposed in the Drugs Controller General of India. After an adverse event, has occurred the investigator is compelled to make a detailed report to the Chairman of the Ethics Committee, the head of the institution where the trial is being conducted and the Licensing Authority within 10 calendar days. In the event of death, the report is sent to the Expert Committee instead of the Licensing Authority. The Ethics Committee is then required to present a report to the Licensing Authority within 21 days after receiving the investigator’s report or to the Expert Committee in the event of death. In cases on injuries that are not fatal, the Licensing Authority makes a ruling within 3 months of receiving the report from the Ethics Committee. After the ruling the Sponsor or person conducting the trial must make compensation and provide the Licensing Authority with details of the payment within 30 days of receiving the ruling.

Failure to pay compensation in terms of the ruling attracts a prison term of 2 years and a fine double the compensation due to the injured person. The sponsor may also be banned from conducting any clinical trials in India. In the event of death, the Expert Committee must make its recommendations on the cause of death and the quantum of compensation to the Licensing Authority within 30 days of receiving the Ethics Committee report, and thereafter the Licensing Authority should make its ruling within 3 months. In the case of death, the Law is crafted in such a way that compensation should be paid within 6 months (10 days for the investigator’s report 21 days for the Ethics Committee to report to the Expert Committee, 30 days for the Expert Committee to make its recommendations, 3 months for the Licensing Authority to decide and 30 days for the sponsor to provide proof and details of compensation.) For all other injuries compensation is paid within 5 months since the process is streamlined to exclude the Expert Committee.

Fourth, per the Law the Ethics Committee makes an opinion on the cause and the quantum of compensation to the Expert Committee; in turn the Expert Committee makes recommendations to the Licensing Authority who in turn makes the decision. The law is silent on how the quantum of compensation is to be determined. It is therefore incumbent upon the Ethics Committees to acquire the skills and expertise to make assessments on compensation.

Furthermore, the Informed Consent Forms should reflect extra information, such as earnings and occupation that assists the Ethics Committees in their assessments. The investigator therefore acquires private information from the participant which may not necessarily have any relevance to the study. This gives rise to a dilemma. On one hand, the Ethics Committees encroach on the privacy of the participants, while on the other hand they need the information in order to determine the quantum of damages in the event of research related injury. This issue therefore needs to be properly canvassed during the informed consent process.

Fifth; the law explicitly catalogues reasons behind injuries that should be compensated. These are:

- Adverse effect of investigational product (s).
- Any clinical trial procedures involved in the study.
- Violations of approved protocols.
- Failure of investigational product to provide intended therapeutic effect
- Use of placebo in placebo controlled trials
- Adverse effects due to concomitant medications excluding standard care
- Injury to children in utero due to parent’s participation in a clinical trial.

The Indian Law applies the “no fault” compensation principle and goes further to include failure of investigational product to provide therapeutic effect as a compensable offence and the use of placebo. This is atypical and may have been informed by concerns that investigational products were being administered disguised as placebos. The pressure on the researchers to report a therapeutic effect may inadvertently introduce interpretation biases [19].

It can be argued that viewed through the prism of the sponsor, the Indian Law is pro- participant, and punitive towards the sponsor, indeed so punitive as to border on the draconian and the immoral. The law, for example, does not take into consideration that in some cases research participants may contribute, through non-compliance or other acts of omission or commission, to their injuries.

Thus, where contributory liability may be appropriate, the law, seemingly oblivious of the fallibility and foibles of the research participant, places the entire burden on the sponsor. This approach smacks of ethical illogicality to the extent that it punishes the sponsor, whose investment in the research enterprise may benefit the society as whole, and rewards the deviant participant, whose actions stand to prejudice the society. However, from the viewpoint of the participants the stringent legal regime helps to offset the massive power differential between powerless participants and very powerful sponsors.

Distinction is not made between participants injured in phase 1 trials who have no direct benefit from the trial and those injured in phase 3 or 4 trials that may be patients whose last hope is in the drug on trial and therefore stand to benefit immensely. Certainly, in phase 3 and 4 trials, the burden to bear the risk is disproportionately thrust on the shoulders of the sponsor. This is blatant violation of the distributive justice tenets. The role of the REC extends beyond prosaic REC roles to include formulating recommendations on the quantum of damages. It can be argued that this departure from orthodoxy is justified in a society where participants are unlikely to independently approach the courts for determination of quantum of damages. This can then be seen not as a new role but just another layer on the REC’s duty to protect the interests of the participants.

### China

In China, clinical trials are regulated by the China Food and Drug Administration and the instrument of regulation is the Provisions for Clinical Trials of Medical Devices (State Food and Administration Agency) SFDA Order 5 of 2004), Good Clinical Practice on Medical Device Clinical Trials (2016) and Guidelines on Ethical Review of Drug Clinical Trials (2010).

. Article 4 of the order states that, “*Clinical trials of medical devices should be conducted in accordance with the ethical principles contained in World Medical Association Declaration of Helsinki, respecting human dignity, striving for maximal benefit for the testing subject, and avoiding harm as best one may.” Article 8 (5) provides that, “Implementer should provide appropriate compensation for the testing subject. The related content of compensation should be described in the clinical trials contract of the medical devices.”* The different guidelines also refer to the Helsinki declaration. However, the different regulations do not make mention of research related injuries at all. It can be surmised that the compensation referred to in Article 8 refers to payments made to research participants as reimbursement for expenses incurred. In 2012 the Ministry of Health made known its intention to put in place a regulation concerning ethical review for biomedical research on human participants. However, extant literature review could not locate such a piece of legislation. In the presence of such a lacuna in legislation, aggrieved research participants may file civil suits in Chinese courts. In doing so they can rely on Article 93 of the Drug Administration Law which states that *“drug manufacturers, drug distributors or medical institutions that violate the provisions of this Law and thus cause harm and losses to users of drugs shall bear the liability of compensation in accordance with law.”* The law does not address the issue of injuries to research participants. For aggrieved participants to receive compensation the medical treatment should be established as the cause of the injury, the process to claim the compensation is often too long and the costs are too prohibitive and often the compensation is too little [20, 21]. Chapter 4 of the Product Quality Law provides for compensation for injuries caused by product defects but defines defect in terms of non-compliance to industry standard. If a manufacturer of a defective product that causes harm or injury can prove that the said product meets the national or industry standard, such a manufacturer would be absolved of any wrong doing. Furthermore, if a drug is approved by the China Food and Drug Administration agency, by definition, it cannot be defective and therefore no claims against it can be made. All the sponsor needs to do is to demonstrate that the investigational product was produced in conformity with industry standards.

Chinese Law therefore does not employ strict “no fault” approach to determination of research related injuries. Injuries not traceable to product quality defects are not covered and, to the extent that the law does not cover the full gamut of research injuries, it appears to be inadequate.

As an alternative to the “no fault approach” Chinese courts have tended to adopt the Equitable Liability Doctrine, whereby a court orders the manufacturer of defective drugs, who may have been deemed not to be at fault, to assume contributory liability and pay part of expenses suffered by the injured participant [21]. A couple of cases are worth noting. In 2006 a woman died 36 days after enrolling on a trial sponsored by Pfizer to test the drug code named “su011248”. The woman started experiencing adverse reactions soon after enrolling on the study. After her death, the husband brought a law suit against Pfizer. The matter took more than 4 years before the Chinese courts. Ultimately the Beijing Number 1 Intermediate Court made an award of $47,460, which Pfizer maintains was contributory liability. This was even though documents before the courts revealed that of the 78 participants enrolled by Pfizer worldwide on the trial, 8 died of adverse reactions shortly after taking the drug. Similarly, in 2000, a patient in Jiangxi Province sued a drug manufacturer after developing encephalitis because of taking the manufacturer’s drug. The court ruled that the drug met industry standards and that the cause of the encephalitis was the patient’s own physical idiosyncrasy. However, the patient was awarded compensation on the Equitable Liability Doctrine to assist him ameliorate the high expenses of coping with encephalitis. The manufacturer was ordered to contribute 30% towards the patient’s medical expenses [21].

### South Africa

The cornerstone of the South African ethical-legal framework for regulating clinical trials is the Department of Health’s Guidelines for Good Practice in the Conduct of Clinical Trials with Human Participants in South Africa (2006) based on the Association of British Pharmaceutical Industries (ABPI) guidelines. This is a very comprehensive framework which catalogues the circumstances and requirements which must be met for injured participants to be compensated. The cardinal provisions of the framework are according to the Department of Health (2006)[22]:

- All participants in clinical trials must be covered by comprehensive insurance for injury and damage.
- The sponsor should pay compensation to patient-volunteers suffering bodily injury, including death but the caveat is that the sponsor’s obligation to pay no fault compensation is without legal commitment.
- Compensation must be paid when on a balance of probabilities, the injury was attributable to the trial or any intervention that would not have been necessary without the trial.
- A child injured in utero due to the participation of the mother in the trial shall be paid as if the child was a patient-volunteer in the trial
- Compensation should be paid only for more serious injury of an enduring and disabling nature
- Compensation should be paid for injuries caused by procedures adopted to deal with adverse events
- The sponsor is under strict liability in respect of injuries caused by the research regardless of the existence or otherwise of negligence by the sponsor
- The sponsor has no obligation to pay compensation for:

1. Failure of medicinal product to have its intended effect or provide any other benefit
2. Injury caused by other licensed products administered to the participant for the purposes of comparison with product under trial
3. Placebo in consideration of its failure to have a therapeutic benefit
4. The extent that injury has been caused by a departure from the agreed protocol; the wrongful act or default of a third party or through contributory negligence (In all these cases compensation may be abated)

- The amount of compensation should be paid in proportion to the severity and persistence of the injury and should be consistent with the quantum of damages commonly awarded by South African courts where legal liability is admitted
- Compensation may be abated or excluded altogether in the following cases:

1. The seriousness of the disease being treated
2. The degree of probability that adverse reactions will occur, and any warning given
3. The risks and benefits of the established treatments relative to those known or suspected of the trial medicines

- Claims should be made to the sponsor by the participant preferably through the investigator
- Claims shall be made for injuries occurring at whatever time from the participation of the research participant in the trial, but not for injuries that may occur due to extended treatment beyond the trial
- The participant has a right to pursue legal remedy in respect of injury alleged to have been caused by the trial
- Participants will normally be asked to accept that payments made under the guidelines are in full settlement of the claims.

For claims to succeed at least 3 requirements should be satisfied. These are conduct, harm and causation. The claimant must demonstrate that an act or omission on behalf of the researchers or sponsors lead to harm (conduct); serious bodily injury of an enduring nature was incurred from a trial product or procedure (harm); and that the harm would not have occurred but for trial participation, and that an unbroken link exists between the conduct and the harm. [21] There is no compensation for temporary pain or discomfort or for less serious or curable complaint

The definition of harm under the South African law is problematic. It takes cognizance of only two characteristics namely, bodily and endurance. This trivializes debilitating injuries that may be transient but more exacting than some long-term injuries. The term “injury of an enduring nature” is also an ambiguity that raises the question-how long is enduring? Is temporary blindness that resolves after 6 months enduring, for example? Or is it so ephemeral that it does not merit compensation? Restricting harm to only bodily injury fails to appreciate the potential virulence of some psychological, social and emotional injuries given the vast amount of HIV, gender violence and other psychological and socio-behavioral research conducted in South Africa.

The prescription that aggrieved participants should preferably make their claims through the investigator fails to take into consideration the possible conflict of interest that may arise because of the symbiotic relationship between the investigator and the sponsor. Indeed, in some cases the investigator may also be the sponsor, thus creating conflicts of interest [23].

The guidelines also permit the sponsor to transfer some of or all his obligations to a contract research organization. If the obligation to pay compensation is not transferred, then the injured participant must seek recompense from the sponsor and this is usually in a foreign court in the case of international multi center research. This can be problematic and prohibitive.

In the case of Venter vs Roche Products and Dr. Gouws and Partners Inc. the court ruled that “by signing the patient information leaflet-informed consent, in which the possible risks of his participation in the trial are fully canvassed, (the) plaintiff accepted the risk of injury by virtue of his participation in a trial. Normally, he would not have the right to claim any damages caused by a trial related injury, as his informed consent constituted a ground of justification for administering of the medication, which could cause him physical harm.” [19, 24] This is contrary to the consensus of bioethicists that informed consent to the risks only authorises that research to proceed, and should not be construed as a waiver of compensation [25].

South African courts are therefore inclined towards viewing a signed informed consent document properly canvassing the risks inherent in the study as absolving the sponsor from the obligation to pay compensation. The provision that compensation is in accordance with ABPI guidelines is also not helpful as “the guidelines recommend compensation without legal commitment, therefore payments which may be made in terms of the guidelines, are to be made *ex gratia.”* The South African guidelines therefore place a heavy burden on the shoulders of the injured participant. The guidelines do not enunciate the role of RECs with regards to compensation for research related injuries.

## Discussion

A comparison of the policies of the BRICS countries reveals that there is a vast panoply of regulations which exist on a continuum in these countries. On one extreme end is India with comprehensive regulations that are codified into law, and on the other end there is China which does not have specific laws regulating research related injuries. There are many differences and similarities.

The first major difference is in the provision of mandatory insurance. Regulations in Russia and South Africa mandate sponsors of clinical trials to provide insurance cover to participants. However, the insurance cover does not indemnify investigators and research institutions. Both the South African regulations and Russian regulations link the quantum of compensation to the severity of the injuries. However, whereas Russian regulations go further to prescribe the amounts to be paid, the South African regulations state that the quantum of compensation should be consistent with awards by South African courts. Brazil and India contain provisions for the compensation for research related injuries in their regulations, but do not require explicit provision of insurance cover. China has no specific law to deal with research related injuries, and aggrieved participants must rely on civil action based on Product Quality laws and Drug Administration laws. Although the requirement to provide compensation is part of the law it is not equivalent to insurance cover.

The second area of major difference is in compensable injuries. Brazil, India and South Africa have regulations that cover both medical treatment and financial compensation over and above the medical expenses. However, in South Africa compensation is for bodily injuries of an enduring nature while in India all injuries are compensable. Furthermore, in South Africa compensation is in accordance with ABPI guidelines which recommend ex gratia payment without legal obligation. Only India provides for the compensation of economic losses. The inclusion of placebos, failure of investigational products to provide intended therapeutic effect and injury from concomitant medications are other unique features of Indian regulations. Russian regulations are silent on medical expenses. South African regulations adopt a “strict liability approach” which closely resembles a no-fault approach. Similarly, India enforces a strict no fault liability approach. This is in contradistinction to the approaches in China, Brazil and Russia where negligence must be established. China is unique to the extent that adherence to industry and national standards exonerates the sponsor from any harm arising from the administration of the products. An ideal approach should impose an ethical obligation on the sponsor to pay for medical expenses, financial compensation for both long term and short-term injuries which should be defined broadly to include both non-physical injuries and compensation for economic losses.

The third area of dissimilarity is in the process to be followed to claim compensation. The India regulations spell out a step by step process with well-defined time lines. The Russian regulations recommend the standard terms and rules pertaining to mandatory insurance cover claims. The South African regulations are unique in recommending that claims should preferably be made through the investigator. Brazilian regulations offer no guidance on how the claims are to be handled which implies that claimants should follow civil court processes. In Brazil, the responsibility for payment lies with the sponsor, the investigator and the institution while in Russia and South Africa payment is from the insurance policy though further legal action is not precluded. In India, the ultimate responsibility is reposed in the sponsor. The Indian regulations facilitate expeditious resolution of claims and should be adopted as the standard.

The fourth sphere of dissimilarity is in the role of the research ethics committees. In this regard India is peculiar in that the regulations require the research ethics committees to provide opinions on the cause of the injury and the quantum of compensation although the final decision is made by the licenser. All other countries’ regulations are silent on this issue. Determination of the cause of injury or injury leading to death requires special skills which may not be in the province of competence of most research ethics committee members. In the case of death; post mortems may be necessary; while in the case of injuries from concomitant medications pharmacology expertise may be required. Determination of the quantum of compensation is even more complex. To execute their mandate research ethics committee members in India need extra skills that are not necessarily required in other countries in the study.

Furthermore, all the countries do not make a distinction between early phase trials involving healthy volunteers and late phase trials with patients. It can be argued that “a same size fits all” approach does not pass the moral test of fairness. Healthy volunteers who have no direct benefit from the medicinal products ought to be compensated better than patients who may be on the trial having exhausted other standard care alternatives. Additionally, patients on trials also benefit from closer attention and monitoring; a benefit not shared by patients on standard care. The Association of the British Pharmaceutical Industry (ABPI) addresses this oddity by recommending differential compensation approaches depending on the phase of the clinical trial.

Of the five countries under comparison India has by far the most comprehensive and most stringent regulations. Distributive justice requires that the benefits and risks accrued in the research enterprise should be equitably carried by the research participants and the society, while compensatory justice dictates that he who has injured has an obligation to compensate the injured part. Often this argument is proffered in advancing the case of the injured subjects. However, in the Indian case, the law requires the sponsor to compensate the participant for injuries arising from concomitant medications as well as for placebos. These are formulations that the sponsor has no control over. The ethical soundness of requiring the sponsor to compensate for injuries arising from placebos and concomitant medications is questionable. The requirement to provide medical treatment for as long as is necessary and provide financial compensation to the same participant could be perceived as “overkill” as some of these participants may require medical treatment until death. Where adequate compensation has been provided, the participant should use this compensation to access medical care. The regulations should explicitly state whether the medical expenses will be covered by the compensation package otherwise the participant may benefit unjustly through double recovery.

Second, while the participants and the society at large arrogate to themselves the benefits of the research all their risks are indemnified by the law. The society bears no risks at all. In other countries, notably France, the law stipulates that the sponsor compensates the participants for injuries suffered in early phase trials where the healthy volunteer does not stand to benefit from the medicinal products, while the society through state funded health insurance, and assumes responsibility for injuries in late phase trials. Placing the burden of compensation excessively on the shoulders of the sponsor through over regulation can have unintended consequences and impede the research enterprise. The number of clinical trials intended to be registered in India fell from 267 in 2010 to 189 in 2012 and 97 in 2013 [26]. The actual number of clinical trials registered declined from almost 500 in 2011 to 19 in 2013 after introduction of the new regulations [27]. In the months following the introduction of the regulations the National Health Institutes (NIH) discontinued 40 clinical trials [15]. Furthermore, the number of new drugs registered in India slipped from 270 in 2008 to 23 in 2013. During the same time India lost its position as the number one destination of clinical trials to China as some sponsors appeared to choose the path of least resistance [27, 19]. If the stringent regulations are diligently enforced sponsors may elect to take their projects to more research friendly destinations. Ultimately, it is the patients who suffer when the research enterprise is impeded. As noted earlier presently there is no consistency in approach towards research related injuries. This lacuna is made worse by the absence of an international best practice. It is therefore important that ethicists should become seized with discussions on formulating an international best practice on compensation for research related injuries. Such a model should make the provision of insurance by sponsors of research studies and the contract organizations, when the conduct of research is contracted out to another party, mandatory. A clear distinction should be made between provision of medical treatment for as long as is necessary and compensation for research related injury. The provision of medical treatment should not in any way detract from the sponsor’s duty to pay compensation over and above the medical treatment. Compensation should be all encompassing and include physical injury, non-physical injury, economic loss and pain. It should also extend to injury in utero. However, an exception should be made for placebos and failure for investigational products to show the intended therapeutic impact. Where injury is caused by the participant’s deliberate non-adherence to protocol the doctrine of contributory negligence and liability should apply as a limit to the blanket no fault compensation approach. This is likely to dissuade the participants from engaging in non-compliance which neither benefits the participant nor the research enterprise in general. Distinction should also be made between compensation in clinical trial phases where the participant has no direct benefit such as phase 1 trials and phase 4 trials where there may be a direct benefit to the participant. The French approach whereby compensation for injury in early phase trials is assumed by the sponsors of the research and compensation for injuries in late stage trials is taken over by the state through a state funded health insurance should be adopted as best practice. The informed consent form and the participant information leaflet should explicitly mention the provision of no fault insurance and spell out the process to be followed to access such claims. This should be comprehensive and should include the relevant timelines as in the Indian regulations. The responsibility to adjudicate compensation claims and to determine the quantum of damages should be vested in a quasi-judiciary entity with experience in determining such matters. The setting up of such an entity should be provided for in the guidelines and the policies.

The prosaic responsibility of the RECs should be expanded to include ensuring that comprehensive insurance for research related injuries is provided for and that the informed consent form and the participant information leaflets do not contain any provisions that might seek to induce the participants to waive their rights to compensation for research related injuries. Furthermore, RECs should ensure that the informed consent forms and the participant information leaflets adequately communicate to the participant the processes to be followed and the relevant contact details of responsible persons in the event of research related injuries.

## Conclusion

Many factors, among them, large numbers of treatment naïve populations, disease prevalence and relatively lower costs, have contributed to make BRICS countries increasingly a destination of clinical trials. However, challenges arise when it comes to dealing with compensation for research related injuries. The court based systems are often too slow, cumbersome and inadequate. It is therefore imperative to enact legislations that protect the participants but also do not stifle the research enterprise. There is need for consistency and even harmonization of such regulations at a global level. A model policy on compensation for research related injuries should borrow from the best aspects of the different policies of the BRICS countries and should be informed by the cardinal ethics principles of autonomy, justice and beneficence.

## Abbreviations

ABPI: Association of British pharmaceutical industries
BRICS: Brazil Russia India China and South Africa
Mesh: Medical subject headings
NIH: National health institutes
OHRP: Office of human research protection
REC: Research ethics committee
SFDA: State food and administration agency
